# Measuring the value of older people's production: a diary study

**DOI:** 10.1186/1472-6963-12-4

**Published:** 2012-01-09

**Authors:** Klas-Göran Sahlen, Curt Löfgren, Håkan Brodin, Lars Dahlgren, Lars Lindholm

**Affiliations:** 1 Department of Public Health and Clinical Medicine, Division of Epidemiology and Global Health, Umeå University, SE-901 85 Umeå, Sweden; 2 Department of Nursing, Umeå University, SE-901 85 Umeå, Sweden; 3 Swedish National Institute of Public Health, SE-831 40 Östersund, Sweden; 4 Department of Sociology, Umeå University, SE-901 85 Umeå, Sweden

**Keywords:** old, production, entitlement, intergenerational fairness, informal care

## Abstract

**Background:**

The productive capacity of retired people is usually not valued. However, some retirees produce much more than we might expect. This diary-based study identifies the activities of older people, and suggests some value mechanisms. One question raised is whether it is possible to scale up this diary study into a larger representative study.

**Methods:**

Diaries kept for one week were collected among 23 older people in the north of Sweden. The texts were analysed with a grounded theory approach; an interplay between ideas and empirical data.

**Results:**

Some productive activities of older people must be valued as the opportunity cost of time or according to the market value, and others must be valued with the replacement cost. In order to make the choice between these methods, it is important to consider the societal entitlement. When there is no societal entitlement, the first or second method must be used; and when it exists, the third must be used.

**Conclusions:**

An explicit investigation of the content of the entitlement is needed to justify the choice of valuation method for each activity. In a questionnaire addressing older people's production, each question must be adjusted to the type of production. In order to fully understand this production, it is important to consider the degree of free choice to conduct an activity, as well as health-related quality of life.

## Background

This article deals with a dilemma in health economics. Healthcare can sometimes improve a person's productive capacity, which is of course a benefit both for the individual and for the rest of society. However, improved productive capacity is usually only valued for persons below normal retirement age. This view is a stereotype - it assumes that people produce up to age 65, after which they only consume. In reality, however, it is reasonable to assume rather that young and old people are heterogeneous, and that some older people may produce much more than we commonly expect. This question has received little attention in health economic guidelines [1]. However, given that more than 17% of the Swedish population are over 65, and that it is well known that people in this age group utilise a large amount of healthcare it is important in Sweden, and similarly in other countries.

After this introduction, which includes a brief review of the concept of "value", this article is organised as follows. The method section describes how diaries were used to collect data from a sample of older people. The results section presents and categorises the activities of the informants, and suggests a value mechanism for each. Finally, the discussion section focuses on how to scale up this research into a larger, representative study.

### Current practice in health economics

A person's age is almost always important in health economic evaluations, as it influences both the size of health gains and, in most cases, the cost of providing care. Today, cost-effectiveness (CEA) and cost-utility (CUA) analyses are the most common evaluation methods. They combine a measure of health gained and resources used (e.g. US$, Euros) and arrive at a cost per health unit gained, applying the normative assumption of health maximisation. Health measures usually combine lifetime lived and a proxy for health-related quality of life such as quality-adjusted life years (QALY) or disability-adjusted life years (DALY).

In health economic evaluations, the societal perspective is normally preferred [2]. This means that all relevant costs and outcomes should be included, regardless of to whom they accrue. The traditional terminology is to divide costs into direct and indirect ones. Direct costs can be both medical and non-medical, but they must relate directly to the intervention under study [2]. Direct medical costs, such as the staff and equipment required to implement the intervention, are not considered in the present article.

Indirect costs are often measured as productivity changes, and are associated with patients' and/or their families' lost ability to work or engage in leisure activities due to morbidity or death [3,4]. An intervention that prevents or restores this loss of ability can account for "indirect savings" which are then subtracted from the intervention cost.

One motivation for including production gains due to an intervention as part of an evaluation is that additional resources are becoming available. The recourses could be added to the healthcare budget and give more QALYs, and thus, better health.

Some criticise the above approach from a human rights perspective, based on the principle that health is a fundamental human right which should not be affected by an individual's productive capacity [5] or age [6]. This view is supported by many ethical declarations. For example, human dignity was underlined in Sweden's parliamentary decision on prioritising health resources; healthcare should be allocated independently of a person's income, age, or social position.

Whether to include productivity costs must be considered an ethical, normative question. It is therefore sensible to involve someone who has the responsibility to make decisions in the public interest, such as politicians, policy-makers, and decision-makers, to set out guidelines in this controversial part of the economic analyses.

To summarise the debate, we feel that those arguing for the inclusion of productivity gains present valid arguments from an efficiency point of view, as do those opposing this view based on arguments of fairness. This ethical dilemma seems to be managed most frequently along two lines: one is to estimate production gains (losses) from a societal perspective only for those included in the labour force, according to market wages; and the other is to undertake the analyses from a healthcare sector perspective only, thereby making the question of production gains irrelevant.

There are very few studies that focus on production in old age and the value of old people's production. To consider this issue does not necessarily mean that we advocate the societal perspective as it is commonly understood, or that we reject the arguments regarding fairness. Rather, we believe the question to be even more important in a more general sense. A growing proportion of the world's populations are retired, with individuals living longer and longer. It is likely that these older people play a vital role in the family and in society; they might take care of their grandchildren, help a disabled neighbour with their garden, take care of their chronically ill spouse, and so on.

### What is known about the production of older people?

Some publicly available statistics can serve as indicators of the degree of older people's participation in the open labour market in Sweden. In 2001, the average exit age from the labour force was 62 years for both men and women [7]. A more recent report by Statistics Sweden stated that 14.7% of 65-69 year-olds and 6.4% of 70-74 year-olds are employed or have their own company, and work 25 hours a week on average; in total, 88 500 older people contribute 2.3 million working hours per week [8].

Informal assistance and care seems to be the most commonly described form of older people's production in Sweden, as well as in other European countries [9]. According to a report by the Swedish government, volunteering and informal help is quite substantial in old age [10]. It is estimated that 56% of men (205 000) and 37% of women (148 000) aged 65-74 years conduct non-profitable work. In terms of time given, the average estimates state 14 hours per month, with slightly fewer hours for women and for the oldest people within the age bracket. According to the same study, 57% of 60-74-year-olds and 22% of 75-84-year-olds provide informal help outside their household. Within their own household, 9% of the oldest provide informal help and caregiving. The gender differences are significant, with 13% of the women and 2% of the men (75-84 years) providing informal help within their household, amounting to an average of 63 hours per month.

To sum up, it seems clear that voluntary and informal work by older people provides a major contribution to the Swedish welfare system. Estimates indicate that informal care contributes significantly to society, equivalent to 120 000-150 000 full-time employees on a yearly basis compared with 110 000-130 000 full-time professionals in medical and social care [11]. However, knowledge is lacking regarding the contribution of other forms of voluntary and informal work by older people, and informal work conducted by the oldest people within this age group.

### How to value all goods including informal care?

The value a person puts on a possession depends on the capacity of that possession to contribute to the fulfilment of the person's goals. It can either have an intrinsic value, or have an instrumental value and thus be considered as a means towards an end. In economics, it is assumed that this value becomes apparent at the point of exchange, when a buyer reveals the most they are willing to give up and a seller the least they are willing to accept. All actors are assumed to be well informed and to have freely chosen to engage in this exchange process. When this exchange is scaled up to a "market", a market price will be established equal to the consumers' marginal valuation of this good. Sometimes the value of older people's production can be directly measured in market prices. For instance, there exist market prices for vegetables, wild berries, mushrooms, and so on. However, for most such production there is no market value. Instead, it is more common to "exchange trades", for example by providing hair cutting services in exchange for snow clearing. This means that many goods and services produced by older people are valued either according to a real market price or as an exchange. In the latter case, the monetary equivalent is difficult to estimate, and the return may simply be gratitude.

The markets for health and social care are different from those of many other goods and services, because of several market failures [12]. The implication is that health and social care cannot be valued within a market or for private exchange. Rather, it is social willingness to pay that decides the value of health and social care; that is, the willingness to pay of public decision makers.

Although volunteer time in health and social programmes is important, its value has to a large extent been disregarded in health economics. There are several methods that can be used [13-15], but there are two main approaches suitable for valuation, which attach value either to the outcome or to the time used as an input [16]. The outcome (or proxy or replacement) method implies that the activities undertaken by the informal caregiver have a market value, namely the price that one would otherwise have to pay if the informal caregiver were unable or unwilling to complete the tasks [14]. The second method is to assign a value to the time used by the informal caregiver, equal to the opportunity cost of the time invested [17]. The two methods would likely yield different results if the caregiver is retired.

Both of the above methods imply that the time spent on informal care is multiplied by a wage rate. The wage can either be the actual forgone wage as a measure of opportunity cost [18], or a market wage for healthcare workers as "replacers" [19]. However, for most retirees the forgone wage is zero or close to zero.

A monetary value of 12.36 Euros per hour for informal care has been derived in the Netherlands [20], while in Sweden the values are 196 SEK (20 Euros) per hour for caregivers with gainful employment and 28 SEK (3 Euros) for others [21]. Other studies use a shadow price for voluntary work and informal care, equal to the price for cleaning work [22]. Irrespective of method used, these rules of thumb are all intended to be a yardstick for valuing informal production gains or losses.

In some situations it is difficult to know where the border lies between informal care and ordinary assistance provided by friends or spouses [23]. The intention in the present study is to go some way towards solving this problem by including the entitlement to health and social care. This is an important but rarely considered foundation for valuing production outputs. Because of this entitlement, we can reasonably assume that society has organised, financed, and provided this service in the case that the informal caregiver is unable to perform the work. In our eyes, this entitlement is crucial in the choice of valuation method; if the entitlement to health or social care is present, it is logical to value the output using the replacement method, otherwise the input valuation (the opportunity cost) should be used.

Sen made a distinction between market-generated (e.g. trade-based) and social security-based entitlements [24], the latter of which has obvious relevance here. One can argue that all human beings have some basic entitlements, such as food and water, shelter, security, and so on. Still, in reality, the entitlements that a person can expect will vary considerably between different countries. The European welfare states are an extreme example, with older people entitled not only to the basic conditions for survival but also to advanced healthcare. In Sweden, people receive an old-age pension when they are 65 years of age. Furthermore, in specific situations, older people have a right to healthcare, home help, a place in a residential home for the elderly, an accommodation allowance, food-box distributions, emergency alarms, transportation services, and in-home healthcare. Another aspect is that in Sweden parents are entitled to stay home with a sick child (0-12 years) with financial support from the government, which becomes relevant when one considers the situation of a grandparent who could provide this care in their place. In other countries, the entitlements regarding health and social care differ. This implies that different value mechanisms must be used for the same service in different settings, depending on the decision-maker's assessment of the entitlement.

### Aim

The ultimate aim in our ongoing research is to value the production done by older people. In this study, the main objective is to identify the kind of information about older people's activities that is both needed to decide on a valuation method and possible to collect by using questionnaires.

## Methods

Theories about how to value goods and services are quite abstract, and need to be operationalised before empirical data can be collected. This process is facilitated if real-life examples, beyond the ones that we can easily imagine from our desk, are available for analysis and discussion.

In this study we aimed to identify both "typical" and "atypical" activities among older people. We believe this is a fruitful way to develop a questionnaire for further empirical data collection on a larger scale. We have attempted to undertake what Charles Ragin described as "interplay between ideas and empirical data" [25]. This process, using both theoretical knowledge and empirical data, is sometimes described as an oscillation between inductive and deductive elements in the research process, and is quite similar to how we generate knowledge in everyday life. We collected empirical data from 23 older people who kept diaries for one week. The study participants were aged between 65 and 87 years and lived in Nordmaling, a small and sparsely populated municipality in the north of Sweden where "everybody knows everybody". The diary method is considered useful for data collection [26-28]. Data were collected in May 2008. The participants were instructed to write down everything they did during one week and to record the start and end times for each different activity. It was stressed that some activities could run parallel to each other (e.g. cooking and caring for a spouse).

The sample of informants was not randomly chosen, as it was our intention to obtain a comprehensive and varied image that would help us deepen our understanding of senior production. With a stratified purposeful sampling method [29], we expected many types of activities to be revealed.

Four key informants with local knowledge were selected by the principal investigator to help choose the participants; they were asked to provide the names of people older than 65 years of age who fit very well to one of the following categories according to the key informants' own interpretation of the different criteria:

• A spouse providing home care.

• A person who manages to take care of themselves but is unable to manage any more.

• A hardworking person or couple.

• A person who helps others, outside the family, with lots of different things.

• A person who helps with tasks related to their grandchildren

• A person who is a member of a voluntary association, involved in several activities during the week.

• A person fitting the proverb: "East, west, home is best; there's no place like home".

An information letter was sent to 31 older people who were identified as described. A physiotherapist, well acquainted in the area, then telephoned each of these people and gave additional information. Eight of the 31 did not want to participate, and the remainder gave informed consent. During the visit the physiotherapist then provided her telephone number and agreed when she would pick up the diaries. For the diaries informed consent were obtained and documented. The study was approved by the Regional Ethical Review Board in Umeå, Sweden (Dnr 08-061 Ö). Having received and read 23 diaries several times, we concluded that we had reached saturation point, in that no new information was provided with the last incoming diaries. We subsequently decided to accept the dropout and end the data collection.

Following the open coding procedure used in grounded theory [30], the information within the diaries was coded and then categorised. Our coding, however, was not unbiased as we focused on the participants' activities within as large a spectrum as possible. Given this interplay between empirical findings and theoretical ideas, we constructed categories in the form of Weberian ideal types [31]; that is, theoretical constructions intended to be compared with empirical phenomena present in reality -- in our case, present in the everyday lives of our sample of older people. Our sorting of activities and their aims into categories or ideal types are presented as well as our analytic distinction between those activities that are part of entitlement and the valuation methods associated with the ideal types. Some codes remained alone and ended up as categories of their own, while other categories included several codes.

## Results

The diaries were completed by 9 men and 14 women. Five informants stated that they had worked less than usual and three more than usual during the one-week study window. The characteristics of the respondents varied considerably.

• All participants were aged between 65 and 88 years, and none of the five-year age groups dominated the others.

• Half of the informants had only elementary school education. Eight of the others had a university degree or similar.

• Most of the informants lived alone (n = 17).

• Half of the group lived in the central part of the municipality, and the rest in small rural villages.

• Most of the informants lived close to their children, though two lived more than 100 km away. Three had no children.

The quality of the diaries was good, with many of the participants providing detailed information. Some wrote short headlines and put them on a time schedule, while others gave long descriptions of each activity, including the specific aim, how often the activity occurred, and their feelings attached to each activity. Some of these activities, such as "caring for memorial stones", were very narrow and were coded accordingly. Other activities, such as cleaning, cooking, and domestic work were grouped together as a first line of categorisation (see Table 1). In total, the activities were classified into 29 different codes.

**Table 1:** Description of activities and number of participants reporting each

	Activities	Occurrences
1	**Writing letters to old friends**	5
2	**Personal administration, e.g**. paying bills	11
3	**Self-care, e.g. a**dministering drugs	4
4	**Fulfilling one's own needs, e.g. shopping and having one's hair cut**	11
5	**Caring for memorial stones**	7
6	**Family activities**	20
7	**Repairs and maintenance of the house (layman level)**	19
8	**Walking others' dogs**	5
9	**Needlework/knitting**	4
10	**Home-help tasks, e.g. assisting with toileting**	12
11	**Household services for others, e.g. caring for children or gardening for others**	28
12	**Looking after one's spouse**	4
13	**Own domestic work, e.g. cleaning or cooking**	6
14	**Escorting others to hospital, dentist etc**.	5
15	**Healthcare provided at home**	4
16	**Visiting a lonely neighbour at home or at a nursing home**	10
17	**Practical work in a non-profit association, e.g. lottery work or baking bread**	10
18	**Participation in activities performed by non-profit associations, e.g. the garden association**	15
19	**Non-profit committee meetings**	14
20	**Participating in further education**	2
21	**Owning a company**	2
22	**Salaried work**	2
23	**Physical activity**	24
24	**Commission of trust, paid by municipality**,	7
25	**Repairs and maintenance work, e.g. painting the windows at home (professional level)**	5
26	**Spending time with friends**	24
27	**Making supportive telephone calls**	8
28	**Gardening at home**	23
29	**Repairs and maintenance outside one's own home**	2
		

This gave us material to construct several "ideal types" [32] that we believe can illustrate the heterogeneity among seniors. Each of these ideal types includes a number of activities that are grouped together and given a distinct hypothetical label.

The first of these types is the "caring human" (A. in Table 2), who provides informal healthcare or home help. A typical case is an old couple where the healthier woman takes care of her husband, who suffers from a stroke or dementia. Without her efforts, professional home help or care in a nursing home would be necessary. Another example is an older person who provides informal care organised by a non-profit organisation. This category includes all activities covered by public entitlement. The valuation mechanism is society's willingness to pay, expressed as the cost of the professional care that would be needed if the informal care was absent.

**Table 2:** Valuation method for the constructed ideal types

Ideal type	Examples (ref to Table 1)	Aims of activity	Part of entitle-ment	Valuation method
**A. The caring human**	Assistance in personal hygiene between spouses or others. 10, 12, 15	Informal healthcare and home help	Yes	Social Willingness to Pay
**B. The retired retired**	Cooking one's own food 2, 3, 4, 13	Decent survival	Yes	Social Willingness to Pay
**C. Non-retired retired**	Owning a company, employment or paid commission of trust 21, 22, 24	Income	No	Agreements within the market
**D. The Good Samaritan**	Helping neighbours with shopping, giving emotional support 1, 8, 14, 16, 27	Mutual support between friends	No	Opportunity cost of time
**E. Active grandparent**	Taking care of grandchildren 11	Support within the extended family	No	Opportunity cost of time
**F. NGO-active**	Work in Red Cross 17, 19	Charity	No	Opportunity cost of time
**G. Ego-active**	Participation in church activities, own physical activities 6, 9, 18, 20, 23, 26, 28	Own satisfaction	No	Opportunity cost of time
**H. Working retired**	Painting neighbours' house, repairing one's own car 5, 7, 25, 29	Income	No	Agreements within the market

Informal healthcare and home help are not necessarily always a free choice; for example, shortcomings in formal care can force a spouse to be an informal caregiver. If home care is the most preferred alternative, it may create process utility beside the outcomes [33]. It is different for the forced caregiver, and this situation may instead decrease utility. In general, it is not easy to be an informal caregiver, with several studies indicating associated health risks [34,35]. The cost of decreased quality of life and/or health may be measured using a quality-of-life instrument

The second category, the "retired retired" (B. in Table 2) are people who under a conventional approach would not be considered to produce anything of value. People in this group typically manage by themselves and, for the moment, do not require societal support. Generally we can assume that these older people choose to take care of themselves; however, there may be situations when this is not a free choice, and is more down to necessity due to lack of services within the community.

The activities of the retired retired are instrumental for a decent and dignified life, and thus are covered by public entitlement. One can argue that public coverage occurs when their capability diminishes, or that these activities performed by capable older people carry a societal value. The latter position can be motivated by two views. On the one hand, the majority appreciate and therefore value the ability for older people to have independent and decent lives. On the other hand, thinking in terms of resources, a great deal of resources can be saved if the need for residential nursing care can be postponed. If this standpoint is accepted, for whatever reason, it means that some preventive activities among the elderly have a societal value, and the mechanism for valuation is thus society's willingness to pay for professional care avoided.

We believe that this view is also applicable to younger people, however, in relation to activities that are instrumental to a reasonable healthy life. Thus, accounting for production within this entitlement, with the individual as both the receiver and consumer, would not change the relations between generations in a CEA; both retired and employed people would be assigned approximately the same production value to be subtracted from the intervention cost.

Some older people do not fully retire. The "non-retired retired" (C. in Table 2) continue with employed work, serve on boards (private or societal), or work in their own company, while the "working retired" (H. in Table 2) continue to perform tasks such as helping neighbours and relatives with repairs and painting the house. The difference between these two ideal types is that the non-retired retired are productive in the regular economy, while the working retired work privately or on the black market. However, both types are valued by agreements on the market.

The "Good Samaritan" (D. in Table 2) also conducts work that must be considered valuable. Activities such as helping neighbours with shopping and providing emotional support are valuable when it comes to quality of life for older people. Despite this, society does not normally consider these activities to be the responsibility of the welfare state, and they are thus not covered by public entitlement. These activities are almost always done by free choice, and are reasonably balanced by increased utility. Subsequently, the production of the Good Samaritan should not be valued using society's willingness to pay, but instead should be measured as the opportunity cost of time.

The category of "active grandparent" (E. in Table 2) covers those active older people who assist the younger generation by collecting grandchildren from kindergarten or taking care of them when parents are busy. This makes it possible for mothers and fathers to work full days, or to work when the children cannot attend day-care or school. There is no societal willingness to pay for this kind of work; however there is significant willingness within the extended family.

A special case within this category is the care of sick children under 12 years. In Sweden, parents are entitled to stay at home with their child in this case, and receive compensation from the social security system. This entitlement can be transferred to other actors such as grandparents, and so a proxy for the value of this kind of senior activity is the parents' gained capacity to produce as a result, the value of which should be shared by the two actors (parent and grandparent).

Many older people work in non-profit organisations such as the Red Cross and pensioners as-sociations, as well as churches; these comprise the "NGO-active" type (F. in Table 2). Activities such as board meetings, lotteries, and fundraising are often a prerequisite for the activities conducted within these organisations. Production done by these older people must be considered to be a result of free choice and should be valued by the opportunity cost of time.

The final ideal type consists of the "ego-active" older people (G. in Table 2), who "only" participate in arranged activities or perform activities in order to enjoy themselves. No entitlement exists, and there is no market value. The cost is equal to the opportunity cost of time, which, in principle, on the margin is equal to the utility gained.

**Figure 1:**
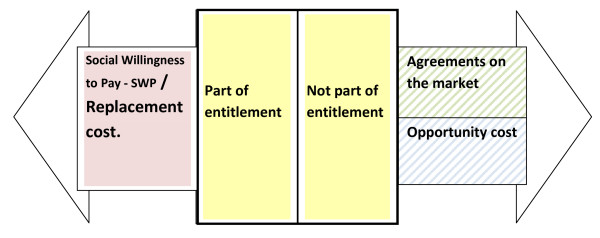
**Older people's production**. Older people's production is either part of or not part of the entitlement. If it is part of the entitlement replacement cost approach should be used. If it not is part of the entitlement agreements on the market or opportunity cost should be used.

The activities, as shown in Table 2 are merged according to their aim. If activities are a part of an entitlement, the valuing mechanism is included in the table. For entitlements, we suggest that the appropriate valuation method is social willingness to pay (SWP); that is, the willingness to pay displayed by public decision makers when allocating resources to healthcare and social care. In many cases, the available information consists of provision costs (the cost to provide the entitlement/replacement cost), and we assume that this cost equals SWP (this is of course a simplification, since SWP can be greater than the cost.) For private goods and services, we suggest a market value. For activities not included in the entitlement or possessing a market value, the opportunity cost of time seems a reasonable measure (Figure 1).

We believe that limitations on a person's right to shape their daily life will seriously decrease quality of life. This is relevant when valuing an activity, since conventional theories assume freedom in choice. We feel that a possible approach when people are illegitimately forced into a particular action is compensation; in other words, that a possible loss of utility could be compensated financially. However, another route is a measure of quality of life, or rather health-related quality of life that may be reduced if an activity involves necessity. We suggest the latter.

## Discussion

We have suggested that a combination of three valuation methods should be used in order to quantify older people's production. However, there are both theoretical and practical problems that should be considered. In the first part of this section we discuss some theoretical aspects, and in the second we discuss some practical problems that arise when taking the next step of attempting to construct a questionnaire with adequate precision.

We have suggested a practical measure of social willingness to pay. If public decision makers decide to allocate resources to a certain service, we simply assume that the value is equal to or greater than the cost. However, this method is not able to quantify a potential consumer surplus. Another option would be to let individuals do the valuation. For most goods, it is assumed that the individual acts as a consumer, and strives for the maximization of their self interest in a more narrow sense. For special types of goods, like healthcare, environmental improvements, and perhaps social elderly care, the self interest model may be less likely. Instead, the individual acts as a citizen, trying to adopt a social perspective [36]. The reasons for this could be pure altruism or a particular form of externality; that we care about our neighbours' health and well-being [13]. We are happy when friends are well-off, and we suffer when they suffer.

Willingness to pay studies in health or elderly care are not a trivial task and methodological reviews have been done [2,37]. One of the major sources of error that occurs is the "embedding" effect, in which the responder has difficulty separating the value of the good itself from the good as a symbol for a more inclusive package [36,37]. We believe this would be a serious problem in willingness to pay studies in the field of elderly care. However, the experience from this particular field is so far very limited, and empirical studies would be necessary to shed more light on these issues.

The opportunity cost consists of the benefits foregone due to spending time on a certain activity. For people engaged in paid work, the benefits can be approximated by the individual's market wage. However, most people in our target group have no market wage. Under these circumstances, it has been suggested that the best approach is to find out the reservation wage [38]. The reservation wage is the rate for which an individual is willing to supply at least one hour on the labour market. While theoretically appealing, in practice this may be somewhat complicated. Firstly, the reservation wage changes on the margin for the same individual; one hour per week requires one rate and 15 hours requires a second rate. The reservation wage even differs between individuals. A second problem is "joint production"; that is, doing more than one activity at the same time. Helping a disabled neighbour to attend a football match, or watching television with one's grandchildren, for example, likely increases happiness for both parties. In these cases, the reservation wage would overestimate the value of benefits foregone.

The opportunity cost of time may be an appealing method of valuation from a theoretical welfare perspective. However, cost estimates based on this measure are seriously limited when the opportunity cost is unknown, which is usually the case for older people. We believe that in many circumstances it is more feasible to value the output, and we suggest this approach when the activities under study are covered by public entitlements. Perhaps the most valuable lesson learned from this pilot study is the importance of an explicit description of the content in an entitlement.

One objection to attempts at valuing older people's time may be that some of the activities portrayed in this study are not particular to older people. Younger and working people may look after their elderly parents, for instance. Is it fair to capture these activities only for older age groups and not for others? It must then be remembered that in cost-effectiveness analyses, the leisure time of people of working age is valued. The assumption is that on the margin, the value of their leisure time is equal to the value of their working time (otherwise they would be working). Hence, the fairness issue in this case concerns the question of how to, as accurately as possible, *also *value older people's time.

In this pilot study we used diaries to gather data, which was a useful method considering our aim, but very time-consuming for both respondents and researchers. Open-ended communication tools such as diaries or narratives need a comprehensive introduction and individual participation in order to obtain data that can be quantified. Analysis also takes considerable time due to the quantity of data collected. In this study, the aim was neither to quantify older people's production nor to address the interaction between or amount of different ideal types. Those aspects remain open for future research.

Possible alternatives to diaries include postal or telephone interviews, which would be particularly useful for a larger representative sample. It has been shown that questionnaires can give rise to a risk of overestimation of the quantity of time [28]. However, if the questionnaire addresses the specific ideal types and very precise forms of production, this risk might be balanced. An interview or questionnaire must include sections for all the ideal type activities presented in Table 2 if we are to obtain a complete picture of older people's production in a wider study. However, some potential participants may feel a heavy "respondent burden", in which case participation in the study would drop, and hence so would data precision. It may therefore be more appropriate to use postal questionnaires. Below, we discuss different sections in the questionnaire. The content of the entitlement is important to justify the choice of valuation method for activities addressed in each section.

One section in the questionnaire is about informal help, care, or support provided within or outside one's household; this covers the ideal type "the caring human" and the linked activities. Societal entitlement and SWP are in focus. It would be necessary to have a record of the hours spent on the associated work, since the valuation would be based on the output and therefore dependent on volume. Questions about health-related quality of life should also be put to both the receiver and the giver of care. Informal care giving may have a negative effect for the giver, particularly if this arrangement is not completely by free choice, and we strongly believe that the dimension of free choice must be investigated in the questionnaire.

Another section of the questionnaire should cover the "working retired" and the "non-retired retired". In order to quantify what is done, questions must be asked about the type and duration of activities. The market price will then be used to measure the production.

Under our definition, the "active grandparent" can enable younger generations to be more productive. This means that we need to better understand how frequently these sorts of support activities occur, because each day carries considerable value. As stressed earlier, this may be an example of joint production in which the value mechanism can be regarded not only as a market price but also as an opportunity cost.

The ideal type "NGO-active" involves quite distinct activities that should be explored in a questionnaire, with particular attention paid to frequency and duration. "The Good Samaritan" is more indistinct or broad and thus more problematic. One solution could be to ask about a number of clearly defined activities within this type.

We are aware of the practical difficulties of using society's willingness to pay for a decent survival in the case of the "retired retired". When professional and informal care can be avoided due to a preventive intervention, one can argue for giving the outcome a value based on the output principal, that is, the cost of home help. It is also possible to argue for using the input principal or the opportunity cost of time, that is, the value of the time spent by the older person to manage by themselves. This discussion is not a matter of vital importance in the context of CEA, however, since the result among retirees and employed people will balance, and therefore not affect the relation between the two groups. We thus believe that this part should not be included in a questionnaire study.

## Conclusions

If a health economic evaluation includes production gains, it is important to take older people's production into consideration. This can be valued either from the input side as the opportunity cost of time or from the output side based on replacement costs. In order to make a reasonable choice between these two methods, societal entitlement is important. One lesson learned from this pilot study is that an explicit investigation of the content of the entitlement is needed to justify the choice of valuation method for each activity. A second lesson is that questions addressing older people's production must be adjusted to the type of production in focus. In order to fully understand older people's production, degrees of free choice to conduct an activity, as well as data describing health-related quality of life, are important.

## Competing interests

All authors declare that they have no competing interests.

## Authors' contributions

All authors have contributed to this manuscript. KGS participated in the discussion of the design and drafted the manuscript. HB. LD and LL contributed in the discussion of the design. LL participated in the design of the article and all authors contributed to finalising the result and discussion sections. All authors have read and approved the final manuscript.

## Pre-publication history

The pre-publication history for this paper can be accessed here:

http://www.biomedcentral.com/1472-6963/12/4/prepub
